# Modularity, balance, and frustration in student social networks: The role of negative relationships in communities

**DOI:** 10.1371/journal.pone.0278647

**Published:** 2022-12-08

**Authors:** José Brito-Montes, Efrain Canto-Lugo, Rodrigo Huerta-Quintanilla

**Affiliations:** Departamento de Física Aplicada, Centro de Investigación y de Estudios Avanzados del Instituto Politécnico Nacional, Unidad Mérida, Mérida, Yucatán, México; Public Library of Science, UNITED KINGDOM

## Abstract

Signed networks provide information to study the structure and composition of relationships (positive and negative) among individuals in a complex system. Individuals, through different criteria, form groups or organizations called communities. Community structures are one of the important properties of social networks. In this work, we aim to analyze the perturbation of negative relationships in communities. We developed a methodology to obtain and analyze the optimal community partitions in nine school networks in the state of Yucatán, México. We implemented a technique based on the social balance theory in signed networks to complete negative missing links and further applied two methods of community detection: Newman’s and Louvain’s algorithms. We obtain values close to Dunbar’s ratio for both types of relationships, positive and negative. The concepts of balance and frustration were analyzed, and modularity was used to measure the perturbation of negative relationships in communities. We observe differences among communities of different academic degrees. Elementary school communities are unstable, i.e. significantly perturbed by negative relationships, in secondary school communities are semi-stable, and in high school and the university the communities are stable. The analyzes indicate that a greater number of negative links in the networks does not necessarily imply higher instability in the communities, but other social factors are also involved.

## Introduction

The field of social network analysis provides a formal way to define important social concepts and study relationships (links or edges) among actors (nodes). In the last years, network analysis has focused on unsigned networks (or networks with only positive links). Signed networks are defined as an extension of networks that include the additional information of positive and negative links. Many social networks allow positive and negative links [1, 2]. In a school network, positive and negative links exist among students, such as our friends and enemies (i.e., ‘hostility’ students), respectively.

Communities in networks are defined as the groups of nodes within which the edges are dense but between which the edges are sparse [3]. Socially, it is expected that people belonging to the same community are more likely to have common hobbies, ideas, etc [4]. Community detection in networks attracts many researchers; thus, many methods have been proposed to study them [5–7]. These methods have been applied with different success rates [8]. The most popular are based on the optimization of a quality function called modularity (proposed by Newman [9]). The extension of the modularity function to signed networks was the first step towards the analysis of community structures in these type of networks [10]. Consequently, the definition of signed communities is an extension of communities definition, by adding ‘as few internal negative edges as possible’ and ‘that these are antagonistic to each other’ [11].

Social balance theory and frustration are two concepts that are linked with studies of signed networks [12]. Social balance is a concept originated in social psychology in the mid-20th century. It was developed by Heider [13] in the 1940s and later adapted to a network-theoretic approach by Cartwright and Harary [14]. For example, states in a triad (three connected nodes), in which the relations of friend-enemy tend to converge to two balanced states: ‘the friend of my friend is my friend’ and ‘the enemy of my enemy is my friend’, and otherwise there will be tension among them [15]. Research based on this theory has been carried out to adress very important issues, such as exchanges of opinion [16], social influence [17], social balance in signed networks, and social balance in signed communities [18]. On the other hand, in signed communities research, frustration is a concept that indicates how far away is a partition of communities from the definition. If communities have many negative links within them or are not antagonistic, frustration will be a high value. A frustration value equal to zero indicates total social stability within the communities (all relationships within them are positive) and that they are totally antagonistic to each other [19].

Properties at the community level may differ greatly from properties at a global network, which makes these studies very relevant to the field. In this work, we use nine real-world student social networks to find optimal community partitions and quantify the perturbation of enmity relationships among students. Consecutively, we raised the question: is the community partition of our networks a reflection of the real partition? We believe that communities that are not significantly perturbed by negative relationships match better communities in the real-system.

The theory of signed communities is under development, most of the proposed approaches have been developed for communities having only positive edges. Our research unites the classical analysis of communities in unsigned networks with the theory of signed communities. We make use of three functions: Modularity, balance, and frustration. In addition, community partitions are analyzed using two methods of community detection: Newman’s (deterministic) [9, 20] and Louvain’s (stochastic) [21] algorithms. These two methods are considered very representative because algorithmically they are different; besides, these are the most used in community theory studies.

Lastly, social interactions in schools have been reported recently and had emerged has an important subject. In reference [22] the authors study temporal evolution in networks obtained using face-to-face interactions among elementary school students using proximity sensors. In particular, friendship networks are also used to study phenomena such as leadership, rivalry, and academic performance among secondary schools students [23]. Also, there are publications where the temporal evolution of the social structure is studied [24, 25].

## Materials and methods

This study was approved by the Bioethics Committee for Research in Human Beings at Centro de Investigación y de Estudios Avanzados del IPN and we obtained written informed consent from the principal of the school that participated in this study. In addition, in elementary schools, the parents/guardians of each child who participated in the survey provided written informed consent. In secondary and high schools, the principals informed parents/guardians who provided verbal consent. On the day of the survey, it was explained to the students that they could decline or give their approval to save their answers. If the student agreed, the student signed an informed consent electronically at the end of the survey.

### Data collection

The data set was collected through the application of surveys (May to October 2019). It is important to clarify that the surveys, although similar, were not applied in the same form. In three elementary schools (esSC, esRRC, and esIZ), due to the unfeasibility of the use of electronic devices, the survey was applied in written form. In the rest of the schools, the survey was electronic, it was conducted using the Internet through a platform that we designed for that purpose. Data collection was supervised personally by professionals in the area, after a brief description of the necessary information for the research. All data was confidentially collected and anonymously analyzed. These data sets have been used in previous research by our work team [26–28].

We designed and applied a questionnaire (survey) to students from nine schools. Table 1 shows the characteristics of the schools relevant to the study. The questionnaire consisted of two parts: the first, with six questions corresponds to general student data, and the second part with seven questions assess data on friendship, enmity, and kinship relationships among students at the same school. We also asked two questions where the students defined what they perceive as friendship and enmity. To avoid conflict or misunderstanding, the term ‘enmity’ was replaced by ‘non-affective relationships’ in the questionnaire. The questionnaire is attached in support information (S1 File).

**Table 1:** Characteristics of the schools that participated in this study.

School	Degree	Age Range	Location	Students
esSC	Elementary	6y to 12y	Rural	108
esRRC	Elementary	6y to 12y	Rural	226
esIZ	Elementary	6y to 12y	Urban	419
ssRDC	Secondary	12y to 15y	Urban	613
ssTN2	Secondary	12y to 15y	Urban	457
ssJLBG	Secondary	12y to 15y	Rural	270
hsCCP	High	15y to 18y	Urban	1497
hsHUN	High	15y to 18y	Rural	74
usTRS	University	18y to 22y	Rural	664

In Mexico, secondary school and high school academic degrees are different and taught in different schools.

### The Dunbar’s ratio

There are several studies in which the friendship relationships are represented by a diagram of concentric circles, where the smaller circles correspond to those friends who are closer to the person being analyzed, that is, in the first circle would be the best friends, in the next one, just friends and so on [29, 30]. It is been observed that there is a ratio among the number of members on each circle. This ratio, called Dunbar’s ratio, is approximately three [29, 31]. Even though this ratio has been observed in several works for the friendship networks, we do not know of any publication in which a similar ratio has been observed in the case of enmity networks. Although some studies describe and analyze varying levels of aggression and hostility between students [32–34], these do not report any ratio among the different levels.

By extracting information from the surveys, the nine corresponding adjacency matrices (*A*) were constructed. Original networks are directed signed networks. We consider that a positive reciprocal relationship between any two nodes (i.e. *A*_*ij*_ = *A*_*ji*_ = 1), is an indication of a strong friendship between students *i* and *j*, which we label a ‘best friend’ relationship. A one-way positive relationship we label a ‘friend’ relationship only. In an analogous way we treat the negative relationship as ‘worst enemy’ if *A*_*ij*_ = *A*_*ji*_ = −1. Just ‘enemy’ if only a one-way negative link exists.

**Fig 1:**
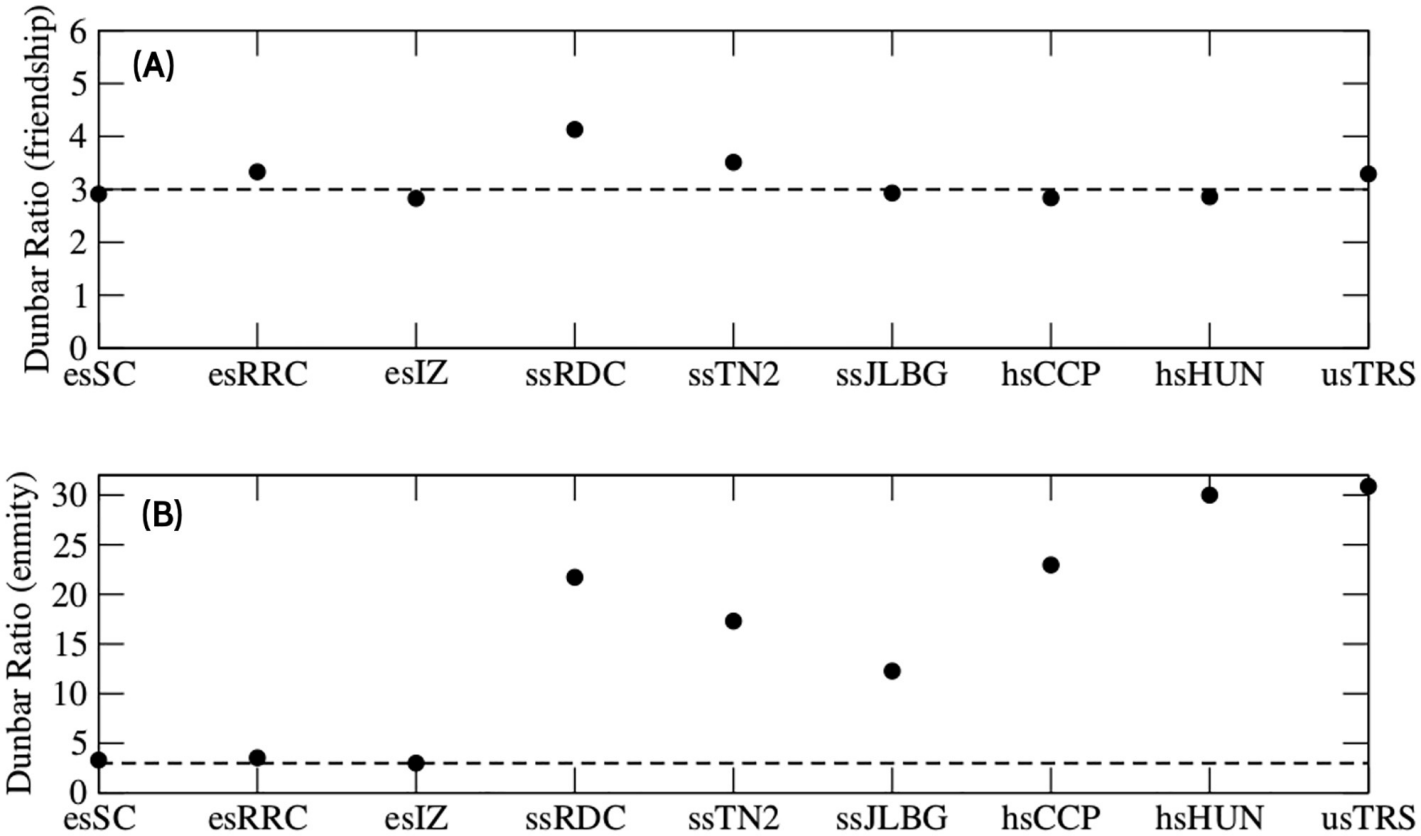
Dunbar’s ratios for experimental data. (A) Values obtained by dividing friend by best friends relationships. (B) Values obtained by dividing enemy by worst enemy relationships. The dashed line is Dunbar’s ratio of three.

What we do next is to quantify the friendship and enmity values (data in Tables A and B in S1 Table, respectively). We graphed Dunbar’s ratios for different schools. We observed that for friendship relationship these values are closer to three (Fig 1A). Similar values are reported in other publications [25]. In the case of enmity relationships, we observed that Dunbar’s ratio was obtained for elementary schools. For the rest of the schools, we get values far from Dunbar’s ratio (Fig 1B).

### Negative missing links

In secondary, high school, and university networks, there was a lack of worst enmity relationship compared to elementary ones (see Table B in S1 Table). This has been observed in secondary schools [25]. This phenomenon was not observed with friendships. We believe that this lack of negative links does not represent the real-system, instead we had incomplete data, and that these can be predicted [12]. There are several obstacles for students to provide us with this type of sensitive information. Although it was explained to them that this information is confidential (since it was an electronic survey in contrast to elementary schools) the participants felt insecure to provide us with information about their enmities in their schools for fear that the information would be improperly accessed. Another reason could be that older students do not provide this type of information as easily as students do in elementary schools.

**Fig 2:**
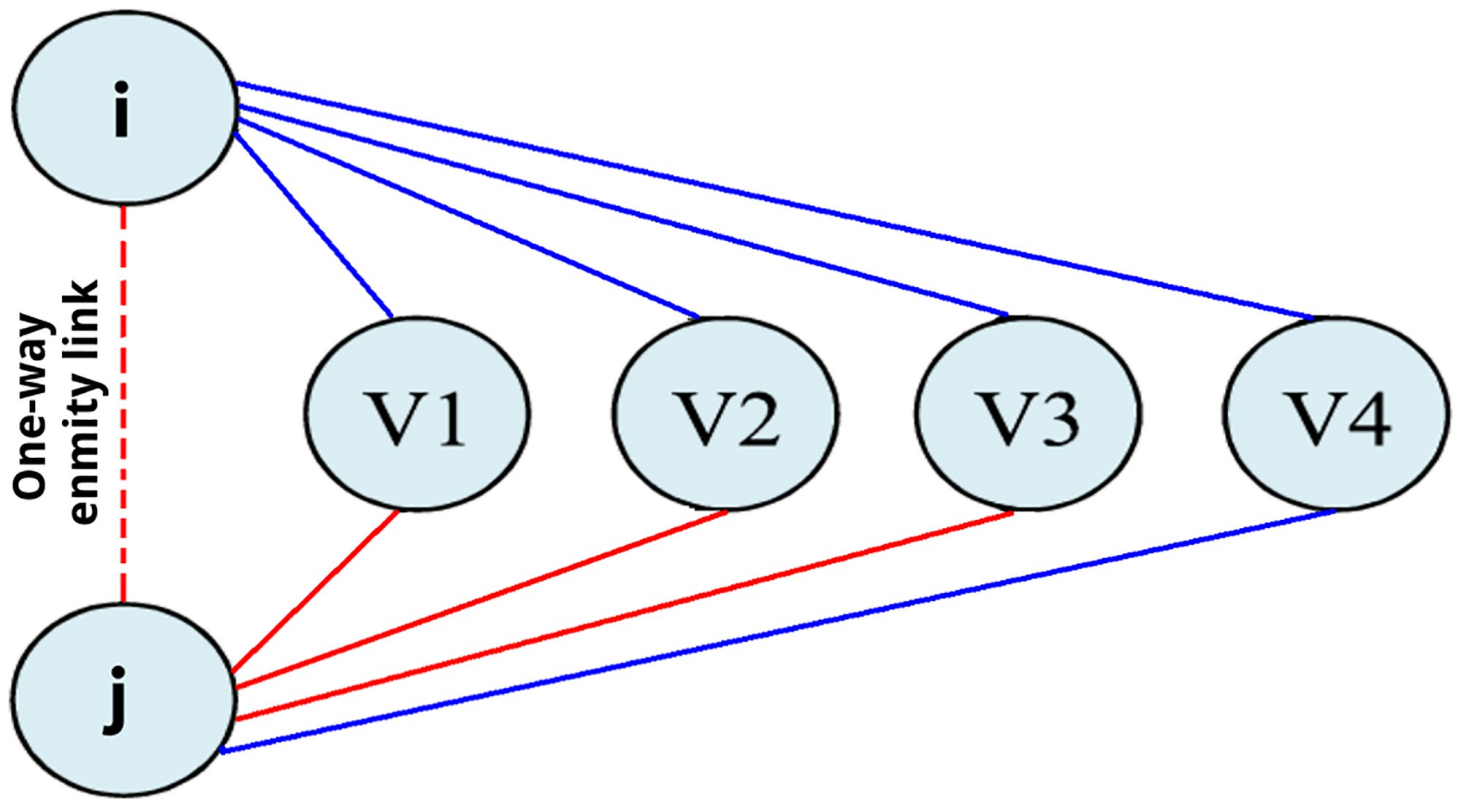
Heuristic to add negative missing links. Blue and red links represent reciprocal friendship (best friend) and enmity (worst enemy) relations respectively. The red dotted line represents a one-way negative link (enemy), that is, only one student said he was an enemy of the other. The figure shows that, if it were reciprocal, there would be three balanced triads and one unbalanced. In this case, the one-way negative link becomes reciprocal negative link. Modified image from [35].

In the following, we present the procedure (heuristic) for adding the negative missing links in the networks (secondary, high school, and university). For this purpose, the social balance theory was used: negative links were added based on the idea that a common enemy of my friends is probably my enemy. To reduce the probability of adding negative links that do not exist in the real-system, a link was added only if one of the two students said he was an enemy of the other, that is if *A*_*ij*_ = −1 or *A*_*ji*_ = −1. In Fig 2 the red dotted line represents a one-way enmity link between nodes *i* and *j*, if this link were reciprocal the network would have three balanced triads ([*i*, *j*, *v*_1_], [*i*, *j*, *v*_2_], and [*i*, *j*, *v*_3_]) and an unbalanced one ([*i*, *j*, *v*_4_]). Since there are more balanced triads, the reciprocal enmity link between nodes *i* and *j* is added.

Table 2 shows the reciprocal positive (*m*^+^) and negative (*m*^−^) links in the networks after applying this methodology. 〈*k*〉^+^ and 〈*k*〉^−^ represent the average degrees of best friend and worst enemy, respectively. Additionally, the *m*^−^ column shows the percentages of reciprocal negative links in the networks. The difference among the node’s column and student’s column in Table 1 is due to the negative of some students to participate in the study.

**Table 2:** Positive and negative links in the signed networks.

Network	Nodes	*m* ^+^	*m*^−^ (%)	〈*k*〉^+^	〈*k*〉^−^
esSC	108	312	141 (31.1%)	5.7	2.6
esRRC	222	553	248 (30.9%)	4.9	2.2
esIZ	417	1403	592 (29.6%)	6.7	2.8
ssRDC	473	583	201 (25.6%)	2.4	0.8
ssTN2	390	629	273 (30.2%)	3.2	1.4
ssJLBG	237	387	183 (32.1%)	3.2	1.5
hsCCP	1429	3025	410 (11.9%)	4.2	0.5
hsHUN	62	95	32 (25.1%)	3.0	1.0
usTRS	561	834	169 (16.8%)	2.9	0.6

Columns *m*^+^ and *m*^−^ represents reciprocal positive and negative links number respectively. 〈*k*〉^+^ and 〈*k*〉^−^ are the best friend and worst enemy links per node.

**Fig 3:**
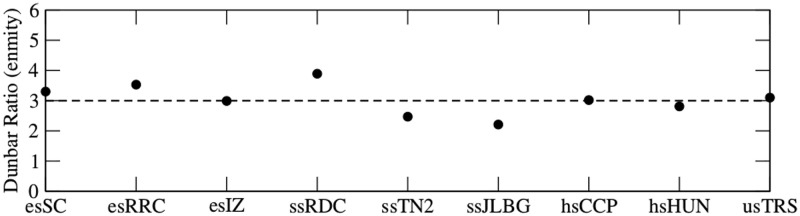
Dunbar’s ratios for negative missing links. Values obtained by dividing enemy by worst enemy relationships. Calculations for secondary and high schools and university were obtained including the missing links. The dashed line is Dunbar’s ratio of three.

After including the missing links (data in Table C in S1 Table), we calculated and graphed Dunbar’s ratios for enmities (Fig 3). We noticed that all values are now closer to Dunbar’s ratio. We consider that this result suggests that our methodology is robust and provides evidence to support our hypothesis regarding negative links. In reference [36], the authors extensively study negative links and the important role they play in the formation and structure of networks. Even though the Dunbar’s ratio has been used in the literature only for positive or friendly relationships, we consider that the present result can shed important light on the subject of negative relationship in signed networks.

We agree with previous works and consider then that friendship and enmity relationships should be reciprocal concepts [37, 38]. From now on, we consider only reciprocal relationships. For this reason, all links that were not reciprocal were suppressed. In this way, undirected signed networks (i.e. symmetrical adjacency matrices) were obtained. Where *A*_*ij*_ = *A*_*ji*_ = 1 indicates a best friend relationship, and *A*_*ij*_ = *A*_*ji*_ = −1 a worst enemy relationship. The nine adjacency matrices are attached in S1 Dataset.

### Newman and Louvain algorithms

Newman and Louvain (greedy) algorithms are two methods of community detection. In both methods, the number and size of the communities are unspecified but determined by the network organization. The aim is to create an optimal community partition on the network; that is, divide it only when a good subdivision exists and leave it undivided otherwise.

The methods use a quality function called modularity (*Q*^+^). It serves to compare the fraction of positive edges found inside communities in the observed network with a similar topological random network. Modularity is defined in Eq 1.

$$\begin{array}{c} Q^{+}=\frac{1}{2w^{+}}\sum_{i}\sum_{j}(w_{ij}^{+}-\frac{w_{i}^{+}w_{j}^{+}}{2w^{+}})\;\delta \left(C_{i},C_{j}\right) \end{array}$$ (1)

where *δ*(*C*_*i*_, *C*_*j*_) = 1 if *i* and *j* nodes belong to the same community, and *δ*(*C*_*i*_, *C*_*j*_) = 0 otherwise. The *w*^+^ symbols represent positive links. Networks with high modularity have numerous connections among the nodes inside a community and few connections among nodes of different communities (see [9] for more information).

The Newman method is a deterministic algorithm that calculates a matrix (named modularity matrix) and diagonalizes it, obtaining its eigenvalues and eigenvectors, these indicate the ‘forces’ with which the nodes belong to one or another community. The aim is to find a partition that maximizes *Q*^+^. A detailed algorithm can be found in ref. [27].

**Fig 4:**
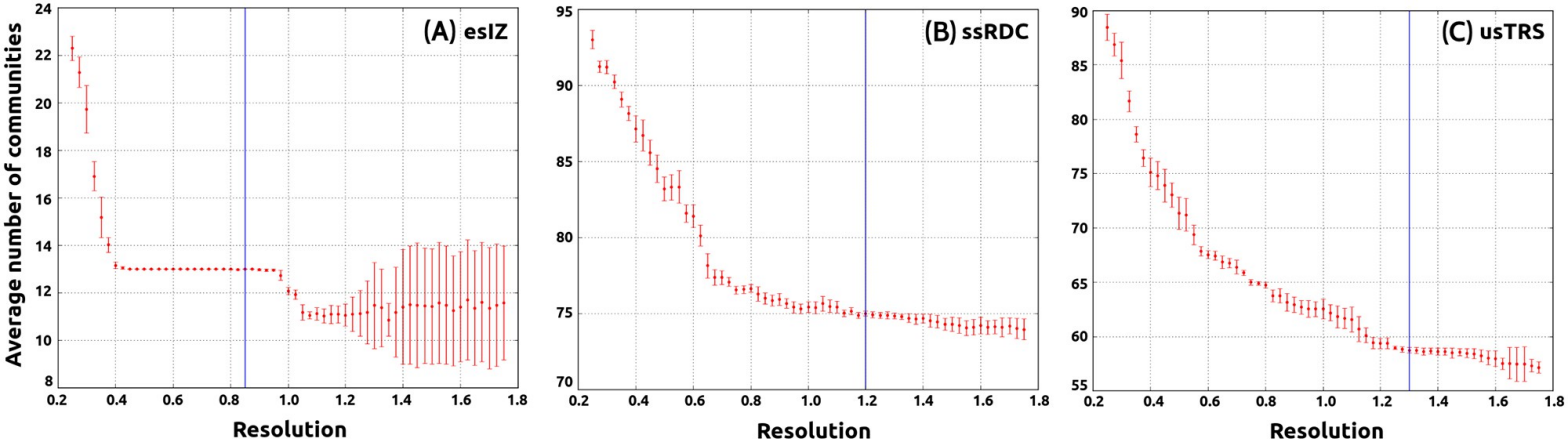
Louvain algorithm calibration. (A) Elementary school esIZ calibration, the optimal resolution value is 0.85. (B) Secondary school ssRDS calibration, the optimal resolution value is 1.20. (C) University usTRS calibration, the optimal resolution value is 1.30. Each value was obtained after 300 simulations.

On the other hand, the Louvain method computes the partition of the network and tries to maximize *Q*^+^. First, it finds small communities by optimizing locally the modularity for all nodes, then each small community is associated with a node and the process is repeated until convergence is reached, details can be found in ref. [21]. Louvain algorithm is stochastic, therefore it generates different optimal partitions in each run. The results obtained from this algorithm must be analyzed as average values. This method has a parameter (named resolution) that must be calibrated (see [39]). Good calibration ensures that the method generates robust and statistically equal optimal partitions. Here, robustness indicates that a small modification of the algorithm does not alter significantly the optimal partition. In Fig 4 the values obtained for the resolution parameter in three schools (esIZ, ssRDC, and usTRS) are shown. The same methodology was applied for all the schools in the study.

Community detection in signed networks is a challenging research problem, which intends to find groups of nodes having positive connections within the same communities and negative relationships between different communities. Several studies have extended the modularity concept to signed networks. Two functions need to be introduced: *Q*^−^ is defined analogously to Eq 1 but for negative links; and *Q*^*s*^ (Q-signed), is a linear combination of *Q*^+^ and *Q*^−^ (see Eq 2). The latter, represents the compensation between the proneness of positive links to form communities and negative links to destroy them [40]. Note that in the absence of negative links *Q*^*s*^ = *Q*^+^.


$$\begin{array}{c} Q^{s}=\frac{2w^{+}}{2w^{+}+2w^{-}}Q^{+}-\frac{2w^{-}}{2w^{+}+2w^{-}}Q^{-} \end{array}$$ (2)

### Social balance and frustration

The social balance is an essential tool for understanding the impact of local interactions on the global structure of signed networks in the context of social relations. This theory considers the possible ways in which triads can be signed. It postulates that triads with three positive links and those with one positive link and two negatives are socially balanced, and therefore should be more frequent in signed networks where the links represent social relationships [2].

We use this concept to determine whether the communities we obtain are socially balanced. Here, the community social balance (CSB) is defined as the ratio of balanced triads in the communities (${△}_{c}^{b}$) divided by the total number of triads within them (△_*c*_), see Eq 3.


$$\begin{array}{c} \text{CSB}=\frac{{△}_{c}^{b}}{{△}_{c}} \end{array}$$ (3)

The frustration concept is based in two ideas. First, the signed community partition should have dense positive intra-connections and as many negative inter-connections as possible. Second, it should have as few as possible negative intra-connections and sparse positive inter-connections. Frustration is the opposite of these ideas, therefore, it is defined as the sum of positive inter-connections plus negative intra-connections, see Eq 4

$$\begin{array}{c} \text{F}\left(C_{1},C_{2},..,C_{k}\right)=\sum_{ij}w_{ij}^{-}\delta \left(C_{i},C_{j}\right)+w_{ij}^{+}\left(1-\delta \left(C_{i},C_{j}\right)\right) \end{array}$$ (4)

where {*C*_1_, *C*_2_, …, *C*_*k*_} is a partition of *k* communities of the signed network. $w_{ij}^{+}$ and $w_{ij}^{-}$ represent the positive and negative links between the communities *i* and *j*, respectively. *δ*(*C*_*i*_, *C*_*j*_) = 1 if *i* = *j* and zero otherwise [19]. We normalize this value concerning the total number of links in the network.

### Signed communities construction

The student social networks that we are studying have two types of links (friendship, and enmity relations) and both kinds of links should be analyzed as a whole thing. In reference [37] the authors study negative relationships and they realized that in a social context, applying standard concepts of social network analysis only to negative links can lead to wrong conclusions. This means that to understand the effect of negative links, one has to consider also the positive ones.

In the present work, we ask ourselves if considering only positive links in the communities analysis, can lead also to some erroneous conclusions. We try to answer this question by implementing the following procedure and quantifying the results. For each of the nine schools, the signed network was built from the friendship and enmity relationships matrix. The positive links subnet was extracted and two community detection algorithms (Newman and Louvain) were applied to obtain the optimal communities partition. Added to this partition are the negative relationships that exist among its nodes; this is how we build signed communities. With this procedure we obtained an optimal partition created from positive relationships containing negative relationships that perturb their stability. The definition of modularity is used to measure this perturbation.

Furthermore, we introduce the concepts of an unstable, semi-stable, and stable community and define them in the social sense. A community that has few antagonistic relationships is expected to have social stability, this means that it will have fewer conflicts among the individuals that integrate it. Whereas a community with many antagonistic relationships will be socially unstable.

## Results

Table 2 shows the properties of the nine networks studied. The proportion (%) of negative relationships (*m*^−^) in elementary and secondary schools is similar, while in high school and university this proportion is lower. It can also be noted that elementary schools have higher values 〈*k*〉^+^ and 〈*k*〉^−^, indicating that in these schools the students have more friendship and enmity relationships than in the rest of the schools. Socially, this could be because at an early age (6y to 12y) students do not have well-defined criteria for selecting their friends, while in adolescence and youth ages (from 12y), they are mature enough to form a better defined social circle. The same phenomenon occurs with enmities.

**Fig 5:**
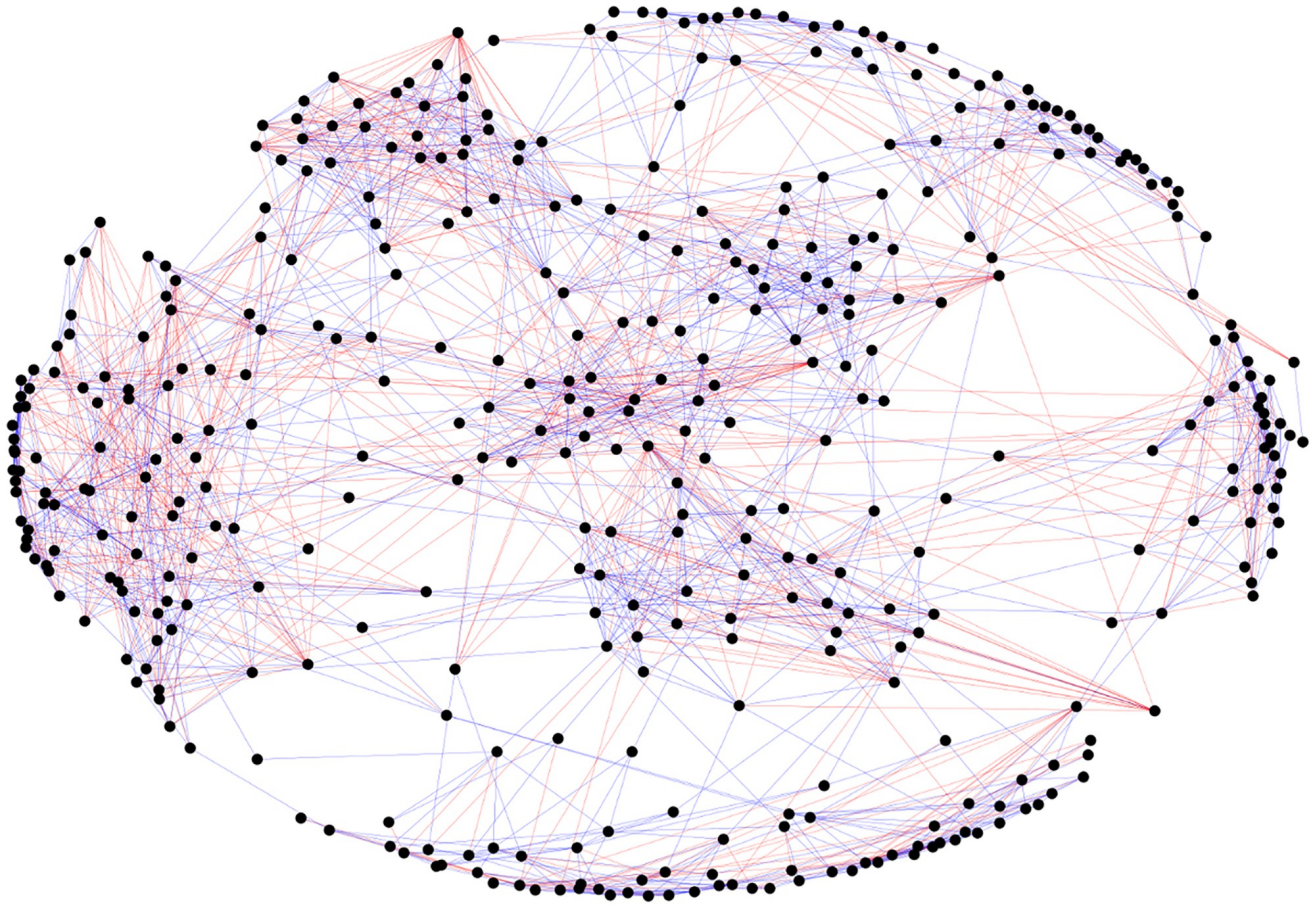
esIZ elementary school signed network. This network has 417 students and 1995 relationships, of which 29.6% are enmity. Each student has on average 6.7 and 2.8 friendship and enmity relationships, respectively.

The high values of 〈*k*〉^+^ and 〈*k*〉^−^ in elementary schools results in the formation of visible groups in signed networks. In Fig 5 shows several groups of nodes from esIZ, which have both positive and negative links, these groups are related to the spatial distribution of students in schools (classrooms). The spatial distribution is important when forming communities since it is very likely that the communities in these schools are mostly made up of students from the same classroom. This phenomenon is not evident in the other schools.

Table 3 show the values of the parameters obtained by applying the Newman and greedy methods. In neither case is there a correlation between the number of communities (Comm) and the school degree. It can be observed that in the greedy method the number of communities is lower for all the cases, as opposed to the Newman method, this is due to the design of the algorithms. There are schools (esSC, esIZ, and hsHUN) where this difference is small, but in other cases (hsCCP, usTRS, and esRRC) the number of the communities is even doubled. The $m_{c}^{+}$ and $m_{c}^{-}$ values represent the number of positive and negative links within communities, respectively. In the greedy method, communities have higher values in $m_{c}^{+}$ and $m_{c}^{-}$, as expected from the large size of the communities. However, contrary to what is expected, in the hsCCP, usTRS, and esRRC schools, the difference of the $m_{c}^{+}$ values between the two methods is not large. On the other hand, the $m_{c}^{-}$ values are more consistent with the community number of each method. The values of the proportion (%) of $m_{c}^{-}$ differ from the those of *m*^−^ values (Table 2): here, elementary schools have higher values than the rest of the schools.

**Table 3:** Positive and negative links in the signed communities.

	Newman method			Greedy method		
Network	Comm	$m_{c}^{+}$	$m_{c}^{-}$ (%)	Comm	$m_{c}^{+}$	$m_{c}^{-}$ (%)
esSC	9	215	23 (9.6%)	6.5	258.3	42.4 (14.1%)
esRRC	16	414	112 (21.2%)	7.8	493.9	166.8 (25.2%)
esIZ	19	1074	325 (23.2%)	13.0	1255.8	462.4 (26.9%)
ssRDC	85	499	19 (3.6%)	74.9	545.8	21.0 (3.7%)
ssTN2	55	480	40 (7.6%)	38.8	574.9	97.0 (14.4%)
ssJLBG	33	338	28 (7.6%)	24.8	363.9	34.7 (8.7%)
hsCCP	80	2089	33 (1.5%)	30.6	2565.7	116.0 (4.3%)
hsHUN	13	71	1 (1.3%)	10.9	81.4	2.8 (3.3%)
usTRS	81	695	17 (2.3%)	58.8	784.8	46.8 (5.6%)

Results of the Newman and greedy methods. Columns $m_{c}^{+}$ and $m_{c}^{-}$ represents positive and negative links within the communities respectively. Each greedy value was obtained after 300 simulations.

**Fig 6:**
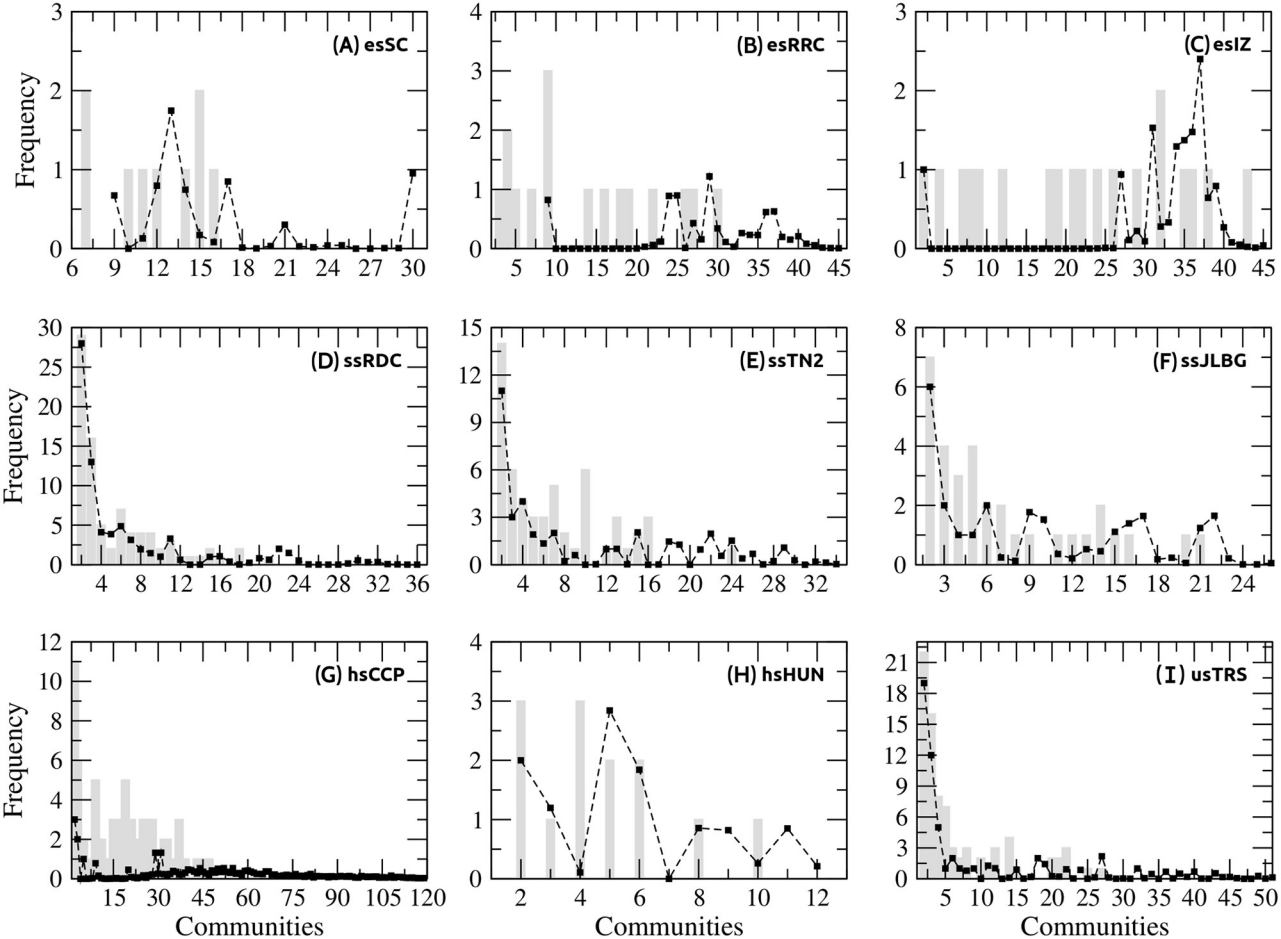
Community distributions. The figure shows the community frequencies in each school. Gray bars for Newman method and dotted lines for greedy method. Each greedy value was obtained after 300 simulations.

The community distributions for each of the schools studied is plotted in Fig 6. Gray bars show distributions from the Newman method and dotted lines distributions from the greedy method. It can be observed in the plot that the greedy method generates communities with more students. For example, in ssRDC school communities are in the range of 2 to 18 students with the Newman method, while with the greedy the range is 2 to 36 students. Furthermore, it can be observed that all the elementary schools, ssJLBG, and hsHUN do not have enough communities to display some distribution pattern. On the contrary, ssRDC, ssTN2, and usTRS schools display an exponential pattern in their community distributions. The distributions of these three schools were compared with the complementary cumulative exponential distribution $e^{-\frac{1}{\lambda }x}$. In all cases, the fitting was greater than 90%, with the values *λ* = 5.51, *λ* = 6.90, and *λ* = 6.82 which represent the average number of students in the community of ssRDC, ssTN2, and usTRS, respectively (Newman method). Similarly, *λ* = 6.31, *λ* = 10.00, and *λ* = 9.55 for the greedy method.

**Fig 7:**
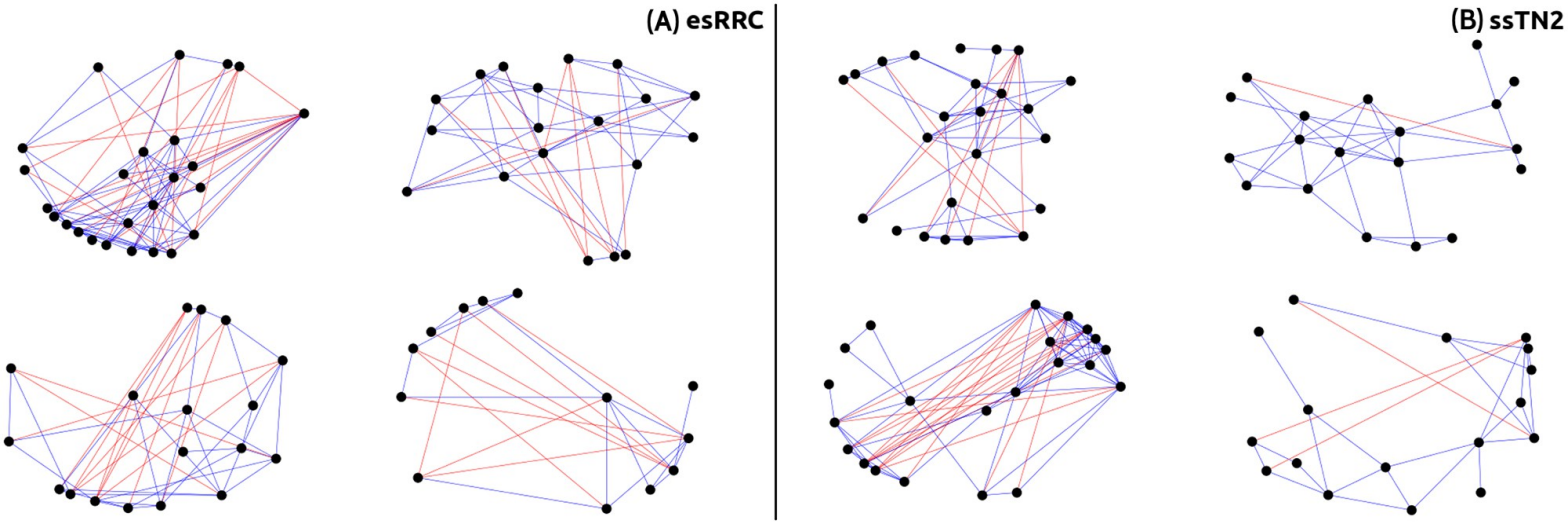
Signed communities examples. (A) Four signed communities from esRRC elementary school (Newman method). Communities with negative hubs and with two antagonistic subgroups are observed. (B) Four signed communities from ssTN2 high school (greedy method). There are phenomena similar to the previous one but in smaller quantity.

We show four communities (Newman) from esRRC school (Fig 7A) and four communities (greedy) from ssTN2 school (Fig 7B). Negative links are observed in both cases. Two phenomena are observed, first, there are specific nodes that perturb the communities, that is, nodes that have many negative links with the other nodes of the same community (negative hubs). It is likely that in the real-system, these students are not part of the communities in which the Newman and greedy methods placed them. The second phenomenon is that there are communities where two antagonistic subgroups of nodes coexist, i.e dense positive intra-connections and dense negative inter-connections. This likely represents two independent antagonistic communities in the real-system. These two phenomena are largely observed in elementary schools and to a lesser extend in secondary schools. High schools and university have a low proportion of negative links in their communities and even some of their communities have only positive links; thus, no significant perturbation.

It would be expected that a small modification to the algorithms solves the problem of perturbed communities. However, this is not so simple, just removing nodes from a community or splitting a community into two causes the modularity function to decrease. Recent studies have addressed this problem through genetic and evolutive algorithms [18].

**Fig 8:**
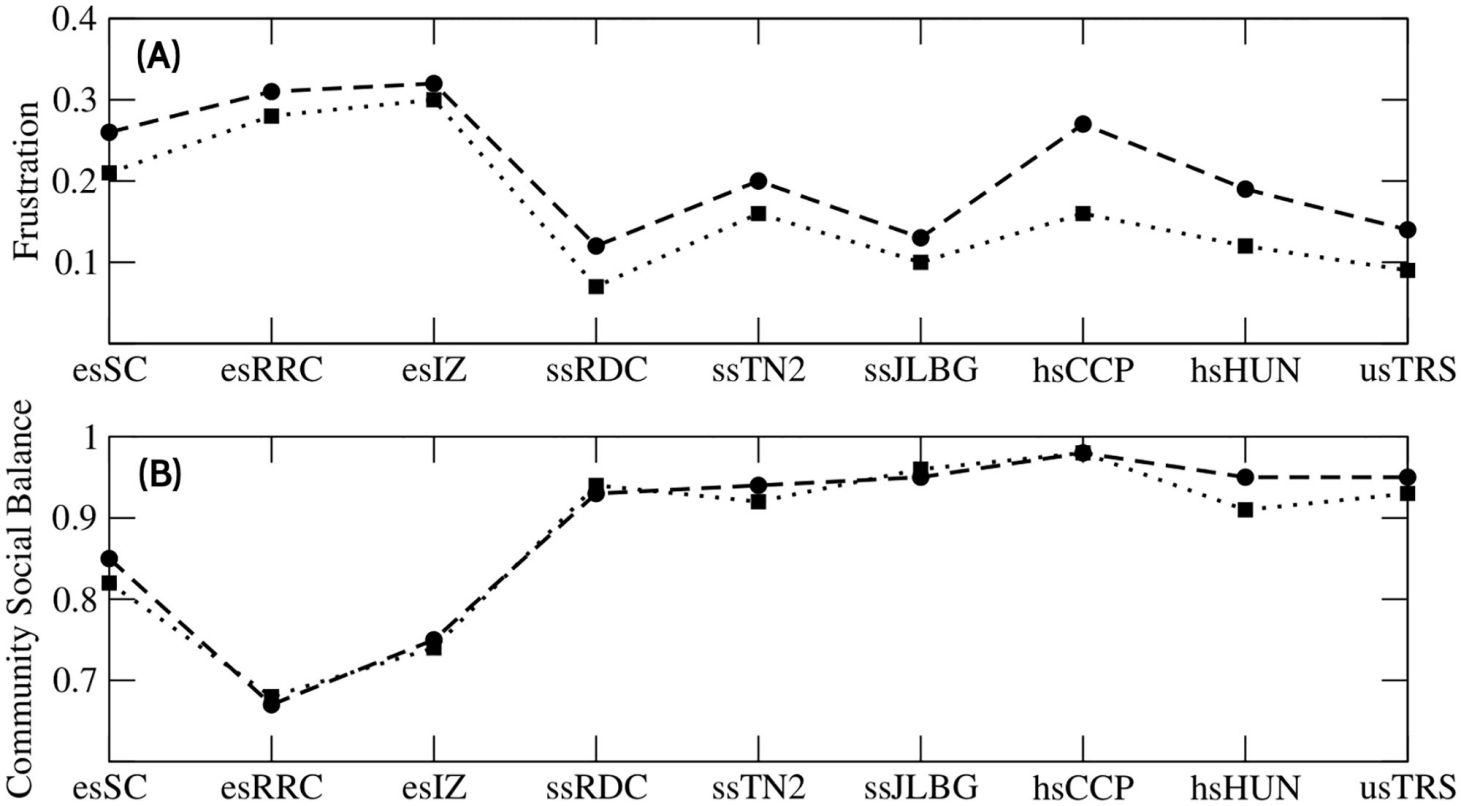
CSB and frustration. (A) Frustration values for Newman (circles) and greedy (squares) methods. (B) CSB (community social balance) values for Newman (circles) and greedy (squares) methods. Each greedy value was obtained after 300 simulations.

In good partitions of signed communities, positive edges should be dense within communities and sparse between communities, and negative edges should be sparse within communities and dense between communities. From Eq 4, it follows that frustration is a function that measures the quality of the partition of signed communities. Fig 8A shows the frustration values of each school. We see that elementary schools have the highest frustration values (in both methods) this is largely due to the number of negative links in their communities. The rest of the schools have lower frustration values.

The signed communities obtained with the greedy method have a higher proportion (%) of negative links (Table 3). However, they have less frustration, this is due to better optimization the number of positive links between communities. This means that in the greedy method there are fewer friendly relations between communities, but they have a greater perturbation of enmity relations (more socially unstable). With the Newman method, the opposite happens.

The difference in the value of frustration between Newman and greedy methods is almost constant (with exception of hsCPP). This is because students in signed networks initially have a fixed number of positive and negative relationships; thus, regardless of the optimal communities partition, the relationships will be the same. This phenomenon may indicate that there is a constant factor that related to frustration with the number of communities in which the school is divided, or more specifically, with their average size.

The CSB is shown in Fig 8B, which indicates the proportion of balanced triads divided by total triads, within community (Eq 3). In elementary schools, the CSB value is lower, which directs to communities with more social tension. In the other schools, the communities have CSB values greater than 0.9, showing no social tension.

It can be noted that the CSB values obtained by both methods are similar (except for hsHUN), this may indicate that the proportion of balanced triads in communities is independent (or nearly) of its optimal partition. This phenomenon is interesting because the CSB values would be expected to be different for different partitions. We believe this is due to signed networks having an intrinsic social balance factor that affects the CSB value. In other words, the number of balanced triads in the network significantly affects the CSB values of any optimal community partition. In conclusion, the social balance factor in systems like the ones we study in this paper provides important information on how balanced their communities are.

**Fig 9:**
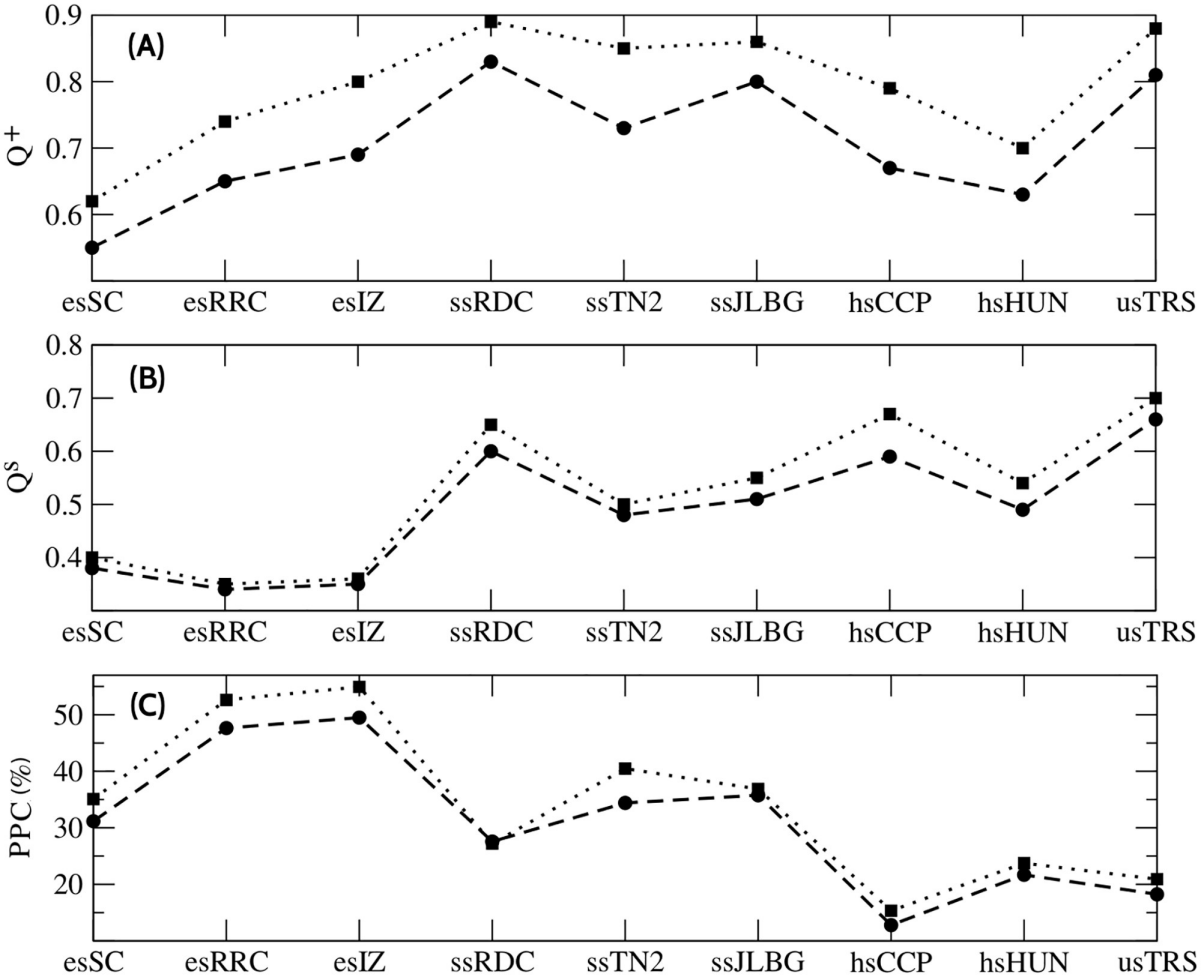
Modularity values. (A) *Q*^+^ values for Newman (circles) and greedy (squares) methods. (B) *Q*-signed values for Newman (circles) and greedy (squares) methods. (C) Percentage of negative links perturbation in the communities (PPC) for Newman (circles) and greedy (squares) methods. Each greedy value was obtained after 300 simulations.

Fig 9A shows the *Q*^+^ values (Eq 1). Theoretically, the range of this value is [−1, 1]. In social systems, this value is usually in the range [0, 1]. High values of *Q*^+^, indicate a better optimization of the partition in communities. It is observed that all schools have high modularity values, e.g. ssRDC, ssJLBG, and usTRS have values close to 0.8 (Newman) and 0.9 (greedy). The latter indicates that the correspondent communities formed by positive relationships meet the community definition very well; i.e. their nodes are densely connected within communities and sparsely connected between communities. In this figure it can be observed that the greedy method optimizes these communities better.

*Q*^*s*^ values (Eq 2) are shown in Fig 9B, with *Q*^*s*^ defined as the difference between *Q*^+^ and *Q*^−^. If a good partition of signed communities exists, the *Q*^+^ value should be large and *Q*^−^ value should be small. *Q*^−^ is the value that measures the perturbation of negative relationships in communities. From the figure, it is evident that some schools are more perturbed than others. Fig 9C shows the percentage of *Q*^−^ perturbation in the communities (Eq 5).


$$\begin{array}{c} \text{PPC}=\frac{Q^{+}-Q^{s}}{Q^{+}}\% \end{array}$$ (5)

An interesting phenomenon is observed: elementary school communities are unstable (i.e. significantly perturbed by negative relationships), secondary schools have semi-stable communities, and in high schools and the university these are stable. We infer that the criteria for choosing relationships and forming social communities of students are better defined over time.

The above indicates that primary school communities are formed with weak criteria (or even random ones). In secondary schools, communities begin to define themselves better and regain a certain degree of stability. Particularly in elementary schools, communities within the real-system could be very different from those generated by the algorithms when we only consider positive links. On the other hand, in the high schools and university, the communities are a very good approximation of the communities in the social system that we study.

## Discussion

Here, we consider the smallest size for a community is two nodes. This consideration initially generated uncertainty because in some networks there are too many two-node communities (see Fig 6) and it is impossible to form triads. To rule out a bias, we tested removing these communities: the results were qualitatively the same.

To better understand friendship and enmity relationships, in the applied surveys, we asked questions about the meaning of these concepts. The responses showed that both concepts have extensive meaning, and include different elements of perception. However, young adults report the greatest consistency in the perception of relationships. Whetnall [41] and Burk [42] report this same result in their research. The better-defined perception of these concepts help students choose their friends better and avoid people they do not like, resulting in more stable communities.

At the beginning of the research, we expected that the PPC value was directly related to the percentage of negative links in the networks, yet this is not the case. Secondary school networks have high percentages of negative links (Table 2), but their communities have low percentages of negative links (Table 3). This indicates two phenomena in these schools: a great selectivity in the formation of their communities; and a behavioral transition from elementary school, to high school and university. This seems to be consistent with the research results of Labianca et al. [43]. Furthermore, Hallinan and Smith indicate that young people become more selective in their friendships as they grow older and demand greater similarity as a prerequisite for friendship [44]. The above explains why, although elementary and secondary schools have the same percentages of negative links in their networks, their PPC values are qualitatively different. Thus, it is evident that a greater number of negative links in the networks does not necessarily indicate greater instability in the communities, but other social factors are also involved. The same happens with the values of CSB and frustration.

The PPC value (Fig 9C) shows a trend concerning the academic grade: except for esSC school, which has a lower value (compared to the other two elementary schools). The esSC is a rural school with a particularity: many of its students are relatives of each other (i.e. siblings or cousins). Generally, kinship relationship implies friendship (this information was obtained from the applied surveys). When esSC students form communities, they integrate their relatives causing more balanced triads (+ + +), and fewer negative links. As a consequence, esSC displays higher CSB value (and less frustration) compared to the other elementary schools (Fig 8B). This phenomenon causes esSC to be an exception to the trend in the PPC value concerning the academic degree. Evidently, esSC is another example that the number of negative links in networks is not directly associated to the PPC value.

The relationship between balance and stability has been studied in the field of signed communities. Sun et al. defined that a community is stable if all the triads in it are balanced [45]. Here, we define the stability of a community concerning the negative links in it (regardless of whether the negative links form balanced triads or not). Fig 8B shows that primary schools (unstable communities) are the most unbalanced. But secondary schools (semi-stable communities) have the same CSB values as schools with stable communities. Therefore, most of the negative links of semi-stable communities are part of balanced triads. The heuristic described in this work adds only negative missing links that have a high probability of existing in the real-system. Nonetheless, it is important to mention that adding these links produces a bias, since the heuristic adds more balanced than unbalanced triads to the networks. Due to this, the CSB values could be overestimated. We calculated the CSB values before the introduction of the negative missing links and we found in all cases values close to 1.0 (data in Table D in S1 Table). The explanation for this is because the negative links are quite rare and many of the triads are of the type (+ + +). This means that when we apply the heuristics we found that the CSB values are lower. Even so, the CSB values could be overestimated with respect to the real values.

We chose two different methods to detect communities. Although the Newman method results in more communities [46, 47], and the Louvain method in larger communities [48], our results are qualitatively the same. The community detection methods are algorithmically different, but they have the same objective: to group nodes densely connected. As a result, communities obtained with different methods are similar. During the algorithmic calculations, we compared the results using the betweenness algorithm [49], and obtained the same conclusions. Regarding the question: Which of the two methods is better? The Louvain method has been demonstrated to be very fast to provide optimal partitions in large networks (its algorithmic order is *O*(*n* * *log*(*n*))) [50]. The networks analyzed in this study are small, thus the difference among the algorithmic execution times was insignificant. On the other hand, the Louvain method generates communities with slightly higher PPC values, but their community partitions have slightly lower frustration values. Moreover, the CSB values are practically the same in both algorithms. For these reasons, in this study, we cannot say which of the two methods is better. To complement our results, we attached in Figs A-E in S1 Fig the boxplots obtained with the Louvian method. Very little variability is observed, which is due to the calibration that was completed as part of the methodology.

Studies on signed communities define that most of the negative links should be observed between communities, with very few negative links within communities. In other words, social communities must be stable. We simulated communities where only positive links were present and obtained communities where the definition of signed communities is not met. We believe that these communities do not represent the real-system. It is not our intention to suggest that the community detection algorithms are erroneous; these methods have been validated in various articles with both, real (one of the best known is the Zachary’s karate club [51]) and synthetic networks, and they show a high-reliability rate. Although, it is known that it is possible to find partitions of Poisson random networks with relatively high modularity values [52] (although they have no community structure). It is possible that the high values of modularity (*Q*^+^) in elementary (and some secondary) school’s communities are the consequence of random relations. The question is, if we have only positive links, how do we know if the modeled communities are a reliable representation? We infer that if individuals have well-defined concepts, we will obtain stable communities and these will be a reliable representation of the real-system.

Negative hubs were found in the communities (see Fig 7). Although exceeds the scope of this study, our methodology may be detecting cases of bullying. These could be either from a community of students to an individual or from a community of students who maliciously integrate an individual to bully him [53]. Helping potential victims and reducing the likelihood of a person being harassed is very important. This is an issue that we are currently investigating.

Finally, there are methods (e.g. machine learning [54]) that allow optimization of signed communities and we believe that the present work can find a way in the implementation of such algorithms to maximize the community’s stability without affecting their modularity. It is also possible to use Eq 2 as an objective function for partitioning the networks into communities [19, 55], and subsequently defining a measurement to calculate the distance between their communities and those obtained in this study. We could approach these problems in a future publication.

## Conclusion

We conducted surveys on friendship and enmity relationships among students from nine schools of different academic grades and built signed networks. A methodology to obtain signed communities and quantify the negative links perturbation in the optimal communities partitions was developed. We obtained Dunbar’s ratios for both types of relationships, positive and negative, and for all the nine schools studied. When analyzing the PPC values, it was observed that a greater number of negative links in the networks does not necessarily imply greater instability in the communities. We also conclude that the academic degree (age) is related to the viewpoint of the concepts of friendship and enmity; and that this influences the stability of the communities.

## Supporting information

S1 File Questionnaires. Questions asked in the data collection (english and spanish versions).
(PDF)

S1 Table Numerical data. Tables with the numerical values of friendship and enmity relationships in school networks.
(PDF)

S1 Dataset Adjacency matrix. Signed adjacency matrices used in the study (.cvs).
(ZIP)

S1 Fig Boxplot for Louvain method. Figures showing the variability of the Louvain method.
(PDF)
