# Supplementary material for: Modularity, balance, and frustration in student social networks: The role of negative relationships in communities
Source: PLoS One. 2022 Dec 8;17(12):e0278647. doi: 10.1371/journal.pone.0278647 (PMC9731467; doi:10.1371/journal.pone.0278647)
Supplement: S1 File — Questions asked in the data collection (english and spanish versions). (PDF) [file pone.0278647.s001.pdf]

SCHOOL NETWORKS SURVEY
English Version

We appreciate your help and your time. We remind you that all the information that you gave us through this media will be treated confidentially and anonymously, way and only will be used for scientific purposes.

Please indicate your username: ______

IMPORTANT NOTICE:

This is not an exam. We are doing a study about friendship relationships of elementary, secondary, and high schools, and universities. We are interested in knowing your opinion regarding your friendly and enmity relationships. Please answer the next questions and if you have any doubt please ask us. Thank you for your help and support. Please remember that the definition of closer friend implies that your friends are people that you do more face-to-face activities like go study, go to play, go to eat, etc. That is, not friends with whom you only have contact through social networks such as Twitter, Facebook, Instagram, etc.

I. Personal Data

1. Please indicate the date: ____
2. Please indicate your age: ____
3. Please indicate your sex: ___ Men ___ Women
4. Please indicate your classroom: ____
5. How many siblings do you have? ____
6. How many cousins do you have? ____

II. Social Data

1. Make a list of words (9 words tops) to indicate what is a friend for you:
2. Make a list of words (9 words tops) to indicate why someone is NOT your friend:
3. Whom of the students of the school are your friends?
4. With which students do you not get along or have a bad relationship?
5. Whom of the students of the school are your siblings?
6. Whom of the students of the school are your cousins?
7. Whom of the students of the school do you usually get along for studying for exams or for doing homework?

To finish the survey, we ask you to please read the next clause and if you accept please write your full name and click on the button 'Agree'. This will be your authorization for us to save your data and use it exclusively for scientific research purposes. If you do not want to share with us the information click the button 'I do not agree'.

Using this media I give my authorization to the Centro de Investigación y de Estudios Avanzados del Instituto Politécnico Nacional CINVESTAV-IPN Unidad

Mérida to save and use all the data that I give them through this web platform. I know that the institution is doing scientific research whose objective is to determine the interaction networks among the students of my school, to understand how information is spread in these networks. I understand that my data are needed for statistical purposes. I had been previously informed about the treatment of my data, which would be treated anonymously and according to the lineaments established by the Federal Institution for Information Access and Protection. I give my authorization knowing the purposes of the research study and the form that all my information is going to be used.

Your full name: ________________________________

AGREE | I do not agree

Spanish Version

De manera anticipada te agradecemos por tu colaboración y tu tiempo. Te recordamos que toda la información que suministres por este medio será tratada de manera confidencial, anónima, y sólo será empleada para investigación científica. Te pedimos llenar el siguiente formulario con total sinceridad y te agradecemos de nuevo por toda tu colaboración y tu buena disposición.

Por favor indica tu número de usuario: ______

AVISO IMPORTANTE

Este no es un examen. Estamos realizando un estudio sobre las relaciones de amistad en los niveles de primaria, secundaria, preparatoria y universitario. Nos interesa conocer tu opinión respecto a tus relaciones de amistad y de tipo poco cordial. Si tienes alguna duda por favor preguntale al encuestador. Gracias por tu ayuda y tu colaboración. Por favor responde las siguientes preguntas de manera sincera. Ten en cuenta que las relaciones de amistad deben ser de carácter personal (personas de tu institución las cuales realices actividades como jugar, comer estudiar, etc), es decir NO amistades con las cuales tengas únicamente contacto a través de redes sociales como Twitter, Facebook, Instagram , etc.

I. Datos Personales

1. Por favor indica la fecha: ____
2. Por favor indica tu edad: ____
3. Por favor indica tu sexo: ___ Hombre ___ Mujer
4. Por favor indica tu curso: ___
5. ¿Cuántos hermanos y hermanas tienes? ____
6. ¿Cuántos primos y primas tienes? ____

II. Datos Sociales

1. Haz una lista de palabras(9 palabras como máximo) que indiquen para ti que es un amigo/amiga:
2. Haz una lista de palabras(9 palabras como máximo) que indiquen para ti por qué alguien NO es un amigo/amiga:
3. ¿Cuáles estudiantes de la escuela son tus amigos?
4. ¿Con cuales estudiantes tienes una relación poco cordial o una mala relación?
5. ¿Quiénes de los estudiantes de la escuela son tus hermanos(as)?
6. ¿Quiénes de los estudiantes de la escuela son tus primos(as)?
7. ¿Con cuáles estudiantes te reúnes para estudiar para exámenes o hacer tareas?

Para finalizar la recolección de los datos lee con atención la siguiente clausula, y si estas de acuerdo, escribe tu nombre completo y haz clic en el botón de 'Guardar' al final de está página. Esto será tu autorización para que guardemos tus datos y los usemos exclusivamente para fines de investigación científica. Si no deseas que hagamos uso de tus datos, por favor haz clic en el botón 'Declinar' al final de la página.

Por este medio doy autorización al Centro de Investigación y de Estudios Avanzados del Instituto Politécnico Nacional CINVESTAV-IPN Unidad Mérida para recopilar

todos mis datos personales suministrados por mí en persona a través de este medio electrónico. Tengo conocimiento de que la institución lleva a cabo un proyecto de investigación científica, cuyo objetivo es determinar las redes de interacción entre los estudiantes de mi escuela para entender como se propaga la información en estas redes. Comprendo que mis datos son necesarios para fines estadísticos. Así mismo, se me ha informado que el tratamiento de mis datos se hará de manera anónima y serán resguardados conforme a lo estipulado por el Instituto Federal de Acceso a la Información y Protección de Datos. Doy mi autorización teniendo conocimiento del fin por el cual han sido recabados.

Nombre completo: ______________________________

GUARDAR   Declinar
