# Supplementary material for: Modularity, balance, and frustration in student social networks: The role of negative relationships in communities
Source: PLoS One. 2022 Dec 8;17(12):e0278647. doi: 10.1371/journal.pone.0278647 (PMC9731467; doi:10.1371/journal.pone.0278647)
Supplement: S1 Table — Tables with the numerical values of friendship and enmity relationships in school networks. (PDF) [file pone.0278647.s002.pdf]

Modularity, Balance, and Frustration in Student Social Networks: The Role of Negative Relationships in Communities.

José Brito-Montes[1], Efrain Canto-Lugo[1*], Rodrigo Huerta-Quintanilla[1].

1 Departamento de Física Aplicada, Centro de Investigación y de Estudios Avanzados del Instituto Politécnico Nacional. Unidad Mérida, Mérida, Yucatán 97310, México.

* ecanto@cinvestav.mx

Table A. Numerical values of friendship relationships in school networks. Experimental data were obtained from the applied surveys. Dunbar's ratios are shown in the final column.

School	Nodes	Friend	Best Friend	Average Friend	Average Best Friend	Dunbar ratio
esSC	108	908	312	16.81	5.77	2.91
esRRC	222	1844	553	16.61	4.98	3.33
esIZ	417	3974	1403	19.05	6.72	2.83
ssRDC	473	2411	583	10.19	2.46	4.13
ssTN2	390	2211	629	11.33	3.22	3.51
ssJLBG	237	1136	387	9.58	3.26	2.93
hsCCP	1429	8593	3025	12.02	4.23	2.84
hsHUN	62	272	95	8.77	3.06	2.86
usTRS	561	2748	834	9.79	2.97	3.29

Table B. Numerical values of enmity relationships in school networks. Experimental data were obtained from the applied surveys. Dunbar's ratios are shown in the final column.

School	Nodes	Enemy	Worst Enemy	Average Enemy	Average Worst Enemy	Dunbar ratio
esSC	108	466	141	8.62	2.61	3.30
esRRC	222	877	248	7.90	2.23	3.53
esIZ	417	1771	592	8.49	2.83	2.99
ssRDC	473	782	36	3.30	0.15	21.72
ssTN2	390	675	39	3.46	0.20	17.30
ssJLBG	237	405	33	3.41	0.27	12.27
hsCCP	1429	1240	54	1.73	0.07	22.96
hsHUN	62	90	3	2.90	0.09	30.00
usTRS	561	525	17	1.87	0.06	30.88

Table C. Numerical values after the negative missing links were added. Third column indicates the percentages of negative missing links added. Final column shows the new Dunbar's ratios for enmity relations.

Schools	Nodes	Neg. missing links added	Average Enemy	Average Worst Enemy	Dunbar ratio
ssRDC	473	18 %	3.30	0.84	3.89
ssTN2	390	31 %	3.46	1.40	2.47
ssJLBG	237	33 %	3.41	1.54	2.21
hsCCP	1429	27 %	1.73	0.57	3.02
hsHUN	62	30 %	2.90	1.03	2.81
usTRS	561	28 %	1.87	0.60	3.10

Table D. CSB values before the negative missing links were added. Third and fourth columns indicate the CSB values for Newman and Greedy algorithms, respectively.

Schools	Nodes	CSB (Newman)	CSB (Greedy)
ssRDC	473	0.99	0.99
ssTN2	390	1.00	0.98
ssJLBG	237	0.98	0.98
hsCCP	1429	0.99	0.99
hsHUN	62	0.96	0.94
usTRS	561	0.99	0.99
