# Supplementary material for: Modularity, balance, and frustration in student social networks: The role of negative relationships in communities
Source: PLoS One. 2022 Dec 8;17(12):e0278647. doi: 10.1371/journal.pone.0278647 (PMC9731467; doi:10.1371/journal.pone.0278647)
Supplement: S1 Fig — Figures showing the variability of the Louvain method. (PDF) [file pone.0278647.s004.pdf]

Modularity, Balance, and Frustration in Student Social Networks: The Role of Negative Relationships in Communities.

José Brito-Montes[1], Efrain Canto-Lugo[1*], Rodrigo Huerta-Quintanilla[1].

1 Departamento de Física Aplicada, Centro de Investigación y de Estudios Avanzados del Instituto Politécnico Nacional. Unidad Mérida, Mérida, Yucatán 97310, México.

* ecanto@cinvestav.mx

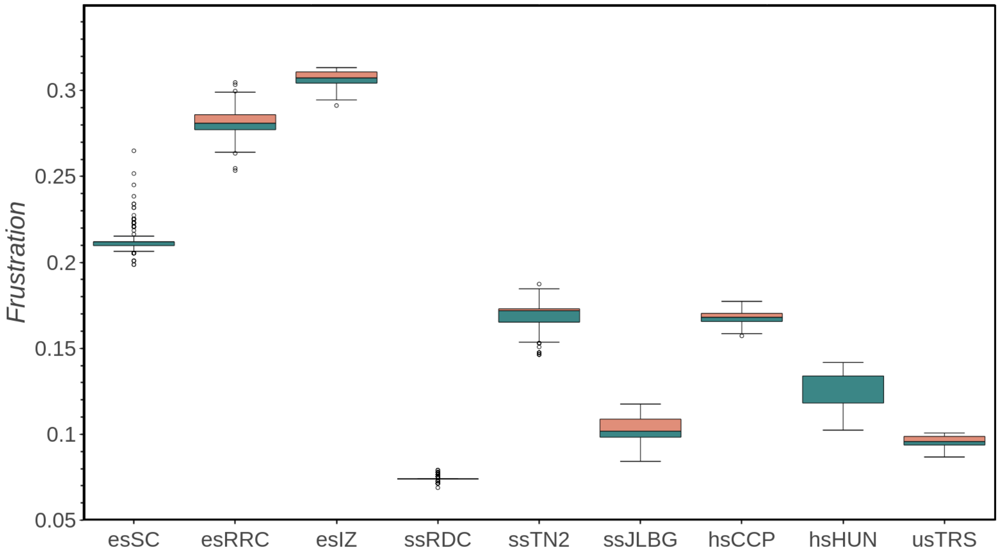

Fig A. Boxplot of the frustration values (Louvain method). Line represents the median, boxes the first and third quartiles, lines, the 95% confidence interval, and dots, outliers. Each Boxplot was obtained after 300 simulations.

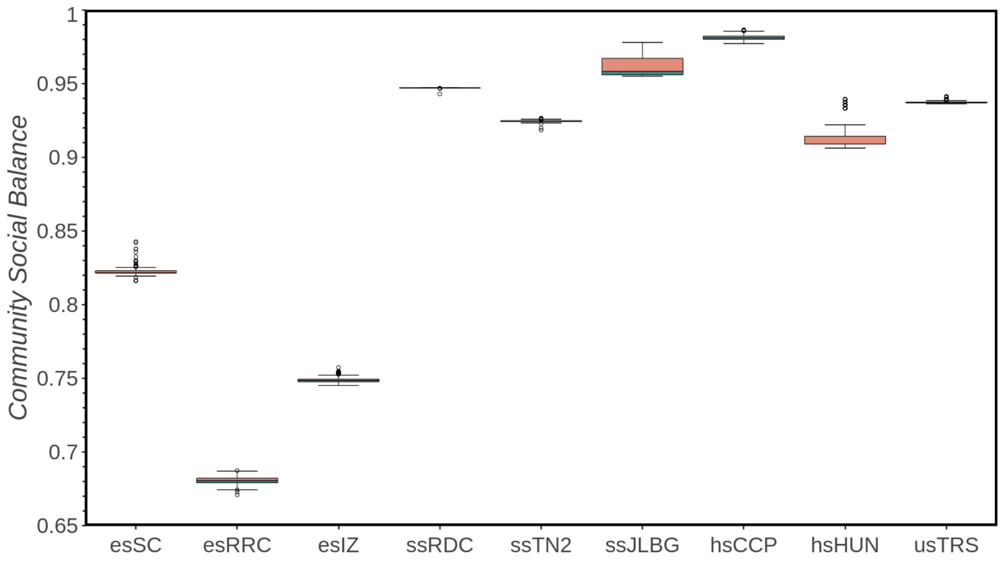

Fig B. Boxplot of the CSB values (Louvain method). Line represents the median, boxes the first and third quartiles, lines, the 95% confidence interval, and dots, outliers. Each Boxplot was obtained after 300 simulations.

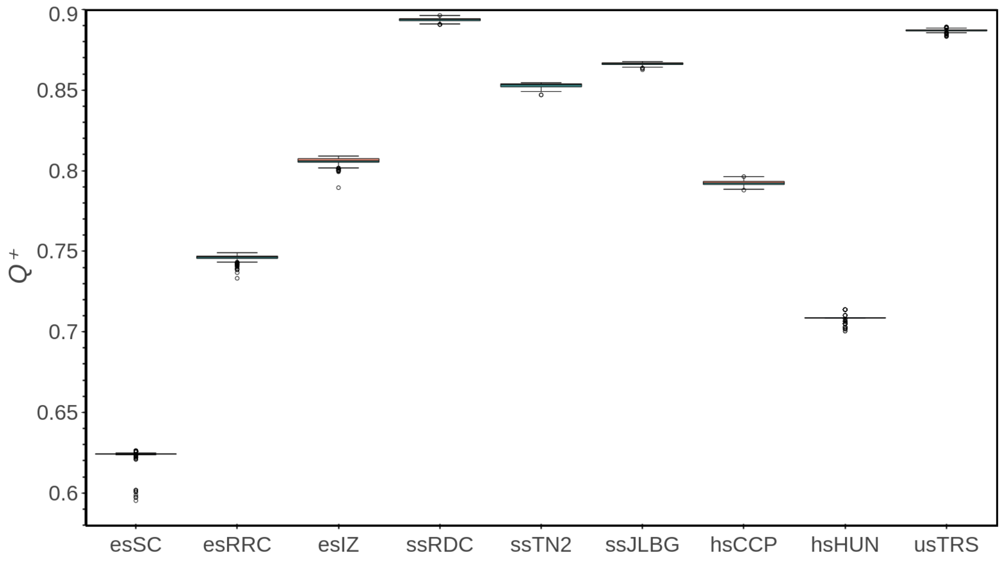

Fig C. Boxplot of the Q^+ values (Louvain method). Line represents the median, boxes the first and third quartiles, lines, the 95% confidence interval, and dots, outliers. Each Boxplot was obtained after 300 simulations.

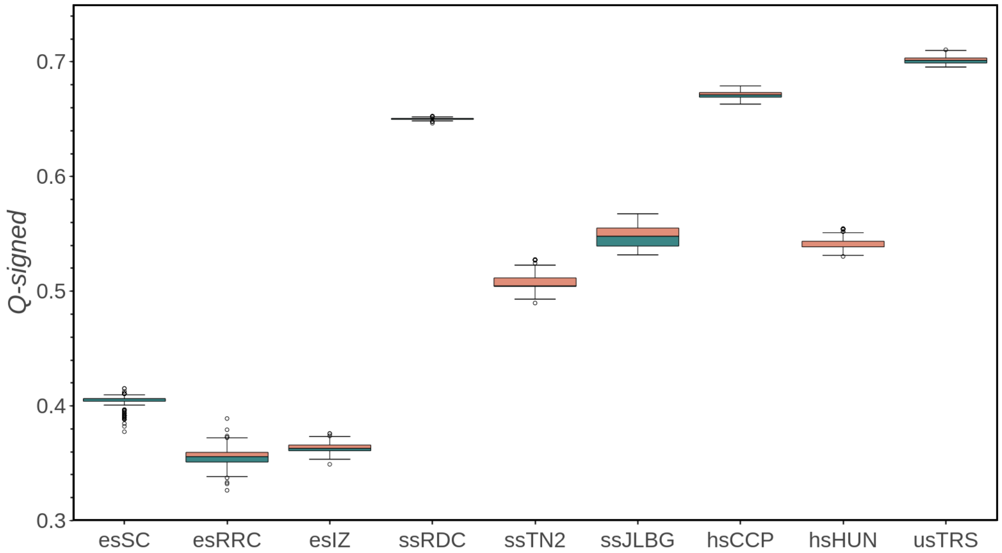

Fig D. Boxplot of the Q-signed values (Louvain method). Line represents the median, boxes the first and third quartiles, lines, the 95% confidence interval, and dots, outliers. Each Boxplot was obtained after 300 simulations.

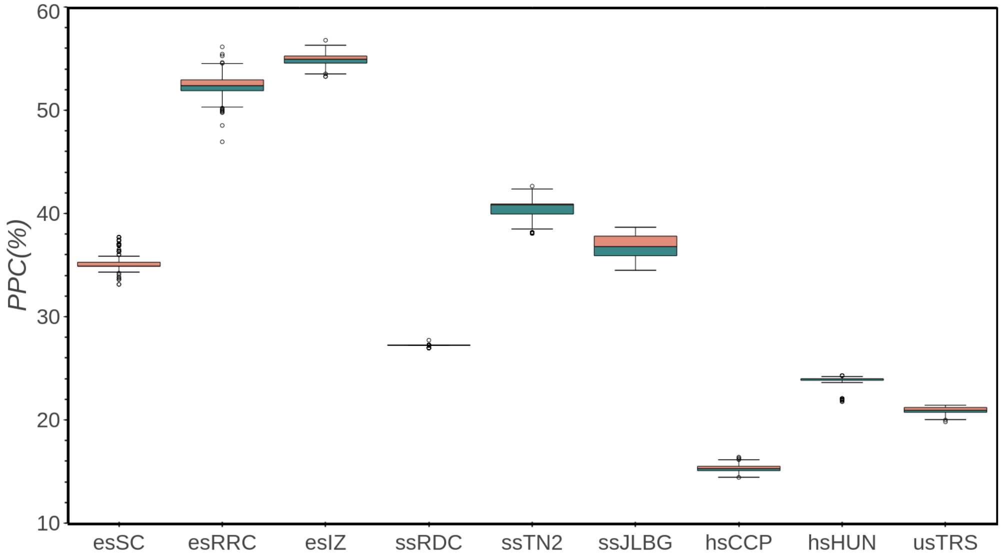

Fig E. Boxplot of the PPC values (Louvain method). Line represents the median, boxes the first and third quartiles, lines, the 95% confidence interval, and dots, outliers. Each Boxplot was obtained after 300 simulations.
